# SRAP analysis on genetic relationships of genotypes in the genus *Malus* Mill.

**DOI:** 10.1080/13102818.2014.948596

**Published:** 2014-10-23

**Authors:** Rongxue Xu, Dechang Hu, Zhongying Chen, Ping Zhang, Xiaoman Jiang, Gengguo Tang

**Affiliations:** ^a^ College of Forest Resources and Environment, Nanjing Forestry University, Jiangsu, Nanjing, P. R. China; ^b^ College of Life Science, Ludong University, Shandong, Yantai, P. R. China

**Keywords:** *Malus* Mill., SRAP, genetic relationship, phylogeny

## Abstract

Thirteen sequence-related amplified polymorphism (SRAP) primers combined with polymerase chain reaction (PCR) were used to evaluate the genetic relationships among 24 genotypes of the genus *Malus* Mill. including Sect. *Malus*, *Baccatus*, *Sorbomalus* and *Chloromeles*. Out of 115 surveyed fragments, 107 (93.04%) were polymorphic. Coefficients of genetic similarity ranged from 0.538 to 0.868, with an average value of 0.720 between pairs of materials, which indicated the high degree of polymorphism within this species. Cluster analysis showed that all genotypes were divided into six groups. A dendrogram showed that some of the clustered genotypes were largely congruent for geographical distribution. The species in Sect. *Malus*, *Sorbomalus* and *Chloromeles* were separated to a great extent. However, the DNA patterns for some genotype groups did not demonstrate relative agreement in their pedigrees. In Sect. *Sorbomalus*, *M. yunnanesis* was independently separated, while *M. prattii* were clustered in group III with *M. bhutanica*, *M. platycarpa* and *M. fusca* classified into Ser. *Kansuenses*. Five species originated from the American region were dispersed into two groups within the dendrogram. The conflicts were reflecting their complex genetic backgrounds.

## Introduction

The genus *Malus* Mill. includes about 43 species of plant germplasm in the world at present. The species’ genetic diversity originates and is centred on China, and is distributed mainly at two centralized locations, Xinjiang and Sichuan-Yunnan-Guizhou regions. There were about 27 wild species of *Malus* including 20 endemic species in China, with distributions held widely in different geographic positions. The status of wild genotypes was reflected by environmental factors and significantly varied. At the same time, the effects resulting from human activities also have their impact. Therefore, the features of these resources had made the mixed distribution of various species possible and gene interpenetration inevitable. Abundant genetic diversity was frequently observed among species, subspecies or variants. Researches on phylogeny and evolution of the genus *Malus* are considered hotspots in the long term. Studies concerning the morphology,[1] cytology,[2] enzymatics,[3] phytochemical properties [4] and other characteristics are still in progress. Random amplified polymorphic DNA (RAPD), simple sequence repeat (SSR) and amplified fragment length polymorphism (AFLP) molecular markers were applied for analysis of the genetic diversity and were used to construct and identify the phylogenetic relationships in the genus *Malus*.[5–7] However, some disputes in the classification system and genetic relationships still exist.

Sequence-related amplified polymorphism (SRAP) developed by Li and Quiros [8] is a technique that uses a set of designed primers to amplify the region-specific open reading frames (ORFs) in a particular gene. These polymorphisms occur due to the different lengths of the introns, the promoter and the spacer region in different individuals and species. Currently, SRAP had been successfully applied to analyse the genetic diversity, to build genetic maps and for cloning of genes.[9–12] The results provided strong evidence for the evolution of different species. Budak et al. [13] concluded that through the SRAP methodology better distinction results and more polymorphic sites could be obtained in comparison to the use of RAPD, SSR and inter-simple sequence repeat (ISSR) molecular markers within *Buchloe dactyloides*. Ferriol et al. [14] addressed that the use of SRAP was more consistent than AFLP in terms of morphology and evolutionary history for *Cucurbita pepo*. Ruiz et al. [15] detected genetic variation sites among tomato varieties by applying the SRAP markers while by SSR such were not identified.

*Malus* species play an important role in breeding, cultivation and germplasm utilization of plant resources around the world and many countries are very concerned about the wild germplasm resources. In order to address the problems related to the scientific area of genetic relationships between plant species and their phylogeny, molecular biology would provide systematic evidence for evolution. In this study the SRAP technique was used to survey the genetic diversity and explore the genetic relationships among 24 genotypes including Sect. *Malus*, *Baccatus*, *Sorbomalus* and *Chloromeles*. The results aimed to provide an important theoretical basis for breeding, protection and utilization of the germplasm resources of the genus *Malus* Mill.

## Materials and methods

### Plant materials in the study

The current study collected plant species representing 24 genotypes of the genus *Malus* Mill. from Yantai Academy of Agricultural Sciences (Shandong, China) Apple Germplasm Repository (Table 1), including 20 species (or subspecies) and 3 varieties. *M. sieversii* had two different genotypes, named as ‘Xinjiangyepingguo’ and ‘Saweishipingguo’.

**Table 1.** The materials used in this study.

No.	Name	Biotype
1	Xinjiangyepingguo	*M. sieversii* (Ldb.) Roem.
2	Maoshanjingzi	*M. baccata* (L.) Borkh. subsp. *mandshurica* (Maxim) Likhonos.
3	Lijiangshanjingzi	*M. rockii* Rehd.
4	Hubeihaitang	*M. hupehensis* (Pamp.) Rehd.
5	Pingyitiancha	*M. hupehensis* var. *mengshanensis* G. Z. Qian
6	Xijinhaitang	*M. sikkimensis* (Wenzig.) Keohne.
7	Bianyehaitang	*M. bhutanica* (W.W. Smith) Phipps.
8	Dianchihaitang	*M. yunnanensis* (Franch.) Schneid.
9	Xishuhaitang	*M. prattii* (Hemsl.) Schneid.
10	Taiwanlinqin	*M. doumeri* (Bois) Chev.
11	Laoshannaizi	*Malus* × astracanica Hort. ex Dum-Cours.
12	Binzi	*M. domestica* subsp*.chinensis* var*.binzi* LY.N
13	Huahong	*M. asiatica* Nakai.
14	Senlinhaitang	*M. sylvestris* (L.) Mill.
15	Daoshengpingguo	*M. sylvestris* var. *praecox* (Pall.) Ponom.
16	Saweishipingguo	*M. sieversii* (Ldb.) Roem.
17	Huaguanhaitang	*M. coronaria* (L.) Mill.
18	Caoyuanhaitang	*M. ioensis* (Wood.) Brit.
19	Bianguohaitang	*M. platycarpa* Rehd.
20	Hehaitang	*M. fusca* (Raf.) Schneid.
21	Zhaiyehaitang	*M. angustifolia* (Ait.) Michx.
22	Shajinhaitang	*Malus* × *sargentii* Rehd.
23	Duohuahaitang	*Malus* × *florbunda* Sieb. ex Van Houtte.
24	Hongroupingguo	*M. pumila var. niedzwetzkyana* (Dieck) Schneid.

### DNA extraction procedures

DNA was extracted using the cetyltrimethylammonium bromide (CTAB) method. The concentration and purity of DNA was determined by using an ultraviolet (UV) spectrophotometer and a 1.5% agarose gel electrophoresis. Total DNA was stored at –20 °C.

### SRAP-PCR

SRAP analysis was conducted according to previously established protocols.[8] In this assay, SRAP primers were synthesized by Shanghai Sangon Biological Engineering Technology and Service CO. LTD. The primer sequences are shown in Table 2. Seventy-seven pairs with primer combinations were obtained based on 7 forward and 11 reverse primers. The polymerase chain reaction (PCR) mixture in a total volume of 25 μL consisted of: 1X PCR buffer, 2.5 mM Mg^2+^, 0.3 mM dNTPs, 0.3 μM primers, 1U *Taq* DNA polymerase (TaKaRa, Japan) and 30 ng of DNA. Amplifications were carried out in a MJ Researsh Thermocycler model PTC-100. The PCR profile was as follows: an initial denaturation at 94 °C for 5 min; 5 cycles of 3 steps: 1 min for denaturation at 94 °C, 1 min for annealing at 35 °C and 2 min for elongation at 72 °C; followed by 30 cycles with an annealing temperature increased to 50 °C, and a final elongation step of 8 min at 72 °C. PCR products were resolved by electrophoresis on 2% agarose gel in 0.5X TBE buffer stained with ethidium bromide. Bands were observed and recorded under UV light with the SYNGENE gel imaging system.

**Table 2.** Primer sequences used for SRAP marker analysis.

No.	Sequence (5′-3′)	No.	Sequence (5′-3′)
Me1	5′-TGAGTCCAAACCGGATA-3′	Em1	5′-GACTGCGTACGAATTAAT-3′
Me2	5′-TGAGTCCAAACCGGAGC-3′	Em2	5′-GACTGCGTACGAATTTGC-3′
Me3	5′-TGAGTCCAAACCGGAAT-3′	Em3	5′-GACTGCGTACGAATTGAC-3′
Me4	5′-TGAGTCCAAACCGGACC-3′	Em4	5′-GACTGCGTACGAATTTGA-3′
Me5	5′-TGAGTCCAAACCGGAAG-3′	Em5	5′-GACTGCGTACGAATTAAC-3′
Me6	5′-TGAGTCCAAACCGGTAG-3′	Em6	5′-GACTGCGTACGAATTGCA-3′
Me7	5′-TGAGTCCAAACCGGTGT-3′	Em7	5′-GACTGCGTACGAATTAGC-3′
		Em8	5′-GACTGCGTACGAATTACG-3′
		Em9	5′-GACTGCGTACGAATTCAG-3′
		Em10	5′-GACTGCGTACGAATTCTG-3′
		Em11	5′-GACTGCGTACGAATTCCA-3′

### Data analysis

A binary matrix reflecting the presence (1) or absence (0) of each band was generated for each cultivar. After SM (simple matching coefficient) was calculated, the unweighted pair group method with arithmetic average (UPGMA) method was used with SAHN clustering program in NYSYS pc 2.10e to construct the dendrogram. The FIND module was used to identify all trees that could result from different choices of tied similarity or dissimilarity values.

## Results and discussion

### PCR amplification

**Figure 1.**
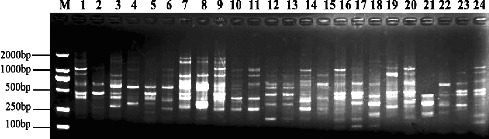
The profile of SRAP amplification using the M7E5 primer combination. M: DL 2000 Marker. The genotype numbers are listed in Table 1.

Out of 77 primer combinations, 13 primer pairs were best in terms of polymorphism level detection and were selected to survey the genetic diversity of the 24 genotypes of the genus *Malus*. The amplification results shown in Table 3 revealed a high level of DNA fragment polymorphisms among the genotypes of *Malus* Mill. A total of 115 nucleotide sequences amplified with 13 primer combinations were observed, whereas 107 fragments were polymorphic (93.04%), ranging between 5 and 13 per primer combination, with an average of 8.85 bands per primer set. The ratio of polymorphism ranged from 71.40% to 100% for each primer combination. The size of scored bands ranged from 150 to 1700 bp. The amplification pattern suggested an abundance of polymorphisms, especially with the primer combination M7E5 (Figure 1). According to the obtained PCR profile, some of the genotypes showed a close relationships and could be distinguished between each other, such as *M. doumeri* and *M. domestica* subsp. *chinensis* var. *binzi*, *M. hupehensis* and *M. hupehensis* var. *mengshanensis*.

**Table 3.** The amplification of SRAP markers on 24 genotypes of *Malus* Mill.

Primer combination	Total number of fragments	Number of polymorphic fragments	The ratio of polymorphic fragments (%)
M1E8	6	5	88.3
M4E7	6	5	88.3
M5E1	11	11	100
M5E2	9	7	77.8
M5E6	7	5	71.4
M5E7	13	12	92.3
M5E8	11	11	100
M5E9	7	7	100
M6E4	7	6	86.0
M6E8	8	8	100
M6E11	6	6	100
M7E5	11	11	100
M7E8	13	13	100

### Cluster analysis

**Figure 2.**
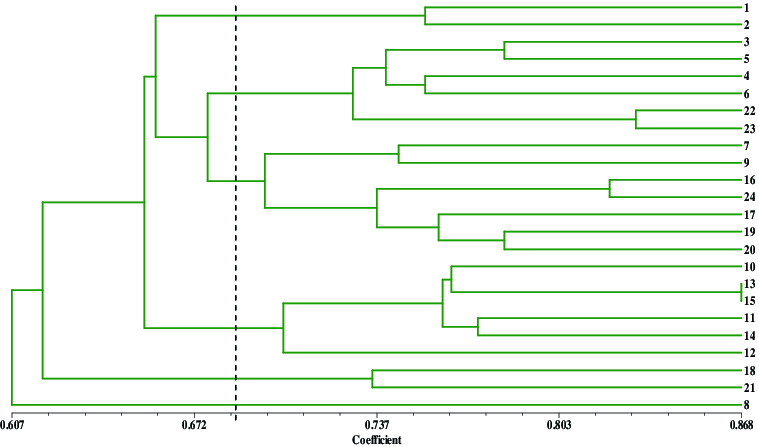
A dendrogram showing the genetic relationships among the 24 genotypes of *Malus* Mill. constructed with unweighted pair-group method with arithmetic averages and based on Jaccard similarity coefficients from the SRAP molecular markers.

The similarity coefficients among the genotypes were computed by using NTSYS- pc2.10 (Table 4) and ranged from 0.538 to 0.868, with an average of 0.720. The highest coefficient between *M*. *asiatica* (No. 13) and *M*. *sylvestris* var. *praecox* (No. 15) was 0.868, showing a close genetic relationship. The lowest coefficient between *M. bhutanica* (No. 7) and *M*. *angustifolia* (No. 21) was 0.538. These similarity coefficients were used to construct dendrograms using UPGMA (Figure 2). Twenty-four genotypes were classified into six cluster groups.

**Table 4.** The similarity coefficients of 24 genotypes of *Malus* Mill. used in this study.

	1	2	3	4	5	6	7	8	9	10	11	12	13	14	15	16	17	18	19	20	21	22	23	24
1	1.000																							
2	0.755	1.000																						
3	0.660	0.717	1.000																					
4	0.575	0.632	0.764	1.000																				
5	0.670	0.745	0.783	0.736	1.000																			
6	0.575	0.670	0.745	0.755	0.717	1.000																		
7	0.679	0.660	0.755	0.670	0.726	0.745	1.000																	
8	0.557	0.594	0.670	0.623	0.547	0.604	0.670	1.000																
9	0.632	0.575	0.613	0.642	0.623	0.736	0.745	0.660	1.000															
10	0.660	0.736	0.642	0.670	0.613	0.708	0.585	0.613	0.764	1.000														
11	0.623	0.623	0.604	0.613	0.575	0.613	0.566	0.575	0.670	0.755	1.000													
12	0.575	0.557	0.594	0.679	0.623	0.623	0.538	0.547	0.604	0.708	0.670	1.000												
13	0.575	0.651	0.670	0.679	0.660	0.679	0.613	0.623	0.679	0.745	0.764	0.774	1.000											
14	0.698	0.623	0.660	0.632	0.594	0.651	0.566	0.575	0.745	0.774	0.774	0.632	0.708	1.000										
15	0.632	0.613	0.670	0.642	0.642	0.642	0.594	0.623	0.679	0.783	0.764	0.736	0.868	0.802	1.000									
16	0.717	0.642	0.642	0.651	0.651	0.632	0.679	0.613	0.745	0.679	0.660	0.651	0.670	0.736	0.764	1.000								
17	0.632	0.689	0.670	0.660	0.642	0.736	0.651	0.566	0.698	0.708	0.670	0.679	0.792	0.689	0.736	0.764	1.000							
18	0.594	0.651	0.594	0.660	0.585	0.604	0.594	0.585	0.585	0.670	0.632	0.604	0.623	0.575	0.585	0.651	0.679	1.000						
19	0.726	0.726	0.670	0.660	0.660	0.679	0.689	0.642	0.755	0.745	0.670	0.660	0.698	0.689	0.736	0.783	0.774	0.698	1.000					
20	0.623	0.660	0.679	0.689	0.632	0.689	0.642	0.651	0.708	0.736	0.698	0.670	0.745	0.717	0.764	0.755	0.745	0.689	0.783	1.000				
21	0.575	0.613	0.594	0.642	0.604	0.642	0.538	0.566	0.547	0.632	0.575	0.642	0.604	0.594	0.623	0.613	0.623	0.736	0.698	0.708	1.000			
22	0.604	0.679	0.774	0.764	0.689	0.726	0.717	0.651	0.670	0.698	0.642	0.557	0.670	0.642	0.689	0.679	0.689	0.594	0.689	0.717	0.613	1.000		
23	0.566	0.660	0.755	0.689	0.689	0.745	0.736	0.613	0.651	0.623	0.566	0.557	0.670	0.566	0.632	0.698	0.726	0.594	0.651	0.642	0.594	0.830	1.000	
24	0.764	0.632	0.670	0.604	0.660	0.623	0.708	0.585	0.698	0.632	0.594	0.585	0.623	0.708	0.698	0.821	0.679	0.623	0.755	0.689	0.585	0.726	0.726	1.000

Group I included ‘Xinjiangyepingguo’(*M*. *sieversii*, No. 1) and *M*. *baccata* subsp. *mandshurica* (No. 2), which were at a similarity level of 0.755. Four wild species native to China: *M. Rockii* (No. 3), *M. sikkimensis* (No. 6), *M. hupehensis* (No. 4) and *M. hupehensis* var*. mengshanensis* (No. 5) were clustered in Group II, together with the Japanese germplasm ‘Shajinhaitang’ (*Malus* × *sargentii*, No. 22) and ‘Duohuahaitang’ (*Malus* × *florbunda*, No. 23). The variant of *M. hupehensis*, ‘Pingyitiancha’, was placed in the same group together with ‘Hubeihaitang’, and the genetic similarity coefficient between them was only 0.768, which may be due to their geographic distant distribution and rich genetic variety.

The genetic backgrounds of the tested genotypes distributed in Group III were relatively complex. ‘Bianyehaitang’ *M. bhutanica* (No. 7) and ‘Xishuhaitang’ (*M. prattii*, No. 9) were *Malus* species from China. ‘Saiweishipingguo’ (*M. sieversii*, No. 16) and ‘Hongrou- pingguo’ (*M. pumila var. niedzwetzkyana*, No. 24) were arranged in one branch at the dendrogram with a similarity level of 0.821. The other three genotypes, *M. fusca* (No. 20), *M. coronaria* (No. 17) and *M. platycarpa* (No. 19), originated from the central parts of America. The dendrogram showed that they had a close relationship between each other. The similarity coefficient between ‘Huaguanhaitang’ and ‘Bianguohaitang’ was 0.774. The exact classification position of *M. bhutanica* has been controversial. While some researchers insisted that *M. bhutanica* was part of the Sect. *Sorbomalus* based on its morphological characteristics,[16,17] Williams [4] considered it closer to the Ser. *Baccatae* according to the results from its flavonoid content, and an SSR analysis that supported the latter view.[18] In this assay, *M. bhutanica* and *M. fusca* were placed together the same group but divided in different smaller clusters. The obtained similarity coefficient was smaller with Ser. *Baccatae*; thus, it was considered by us that *M. bhutanica* should belong to Ser. *Kansuensis* in Sect. *Sorbomalus*, which was also consistent with the hypothesis of Shi et al. [6] based on results from AFLP markers.

Group IV consisted of the genotypes ‘Laoshannaizi’ (*Malus × astracanica*, No. 11), *M. doumeri* (No. 10), *M. domestica* subsp*. chinensis* var. *binzi* (No. 12) and *M. asiatica* (No. 13), which were species cultivated in China. *M. sylvestris* (No. 14) and *M. sylvestris* var. *praecox* (No. 15) were also placed into this cluster. All genotypes in this group were classified to the Sect. *Malus*. These four cultivated species (No. 10–No. 13) originated in China and were clustered into Group IV, once again an indication for their close genetic relationships. Two accessions, *M. ioensis* (No. 18) and *M. angustifolia* (No. 21) originated from Central America and formed Cluster V with a similarity level of 0.652. The genotype *M. yunnanensis* (No. 8) formed an independent group VI. As an ancient diploid species native to China, *M. yunnanensis* has its original characteristic features significantly different compared to the patterns of the other analysed materials. These results are also in agreement with an isozyme analysis showing that the species of Ser. *Yunnanensis* in the *Malus* genus were more primitive.[19]

## Conclusions

In this study, the genotypes with same parental blood were clustered together, and their similarity coefficients were higher, also reflecting the close relationship between them, such as with *M. hupehensis* var. *mengshanensis* and *M. hupehensis*, ‘Shajinhaitang’ and ‘Duohuahaitang’, etc. The constructed dendrogram showed that some of the clustered biotypes were largely congruent in relation to their geographic distribution. The genotypes in Sect. *Malus*, *Sorbomalus* and *Chloromeles* were separated to a great extent. However, the DNA patterns for some biotype groups did not demonstrate a relative agreement in their pedigrees. In Ser. *Yunnanensis*, *M. yunnanesis* was independently separated, while *M. prattii* was clustered in Group III with *M. bhutanica*, *M. platycarpa* and *M. fusca* classified into Ser. *Kansuenses*. Five biotypes that originated from the American Region were dispersed into two groups within the dendrogram. These conflicting observations reflect their complex genetic backgrounds. A follow-up research that includes combination with SSR, ISSR and other molecular markers can be carried out. Through a further increase in the number of markers and expansion in the scope of biotypes, a vaster theoretical basis addressing the *Malus* resources, genetic relationships and taxonomic status would be provided.
